# Early-Life Arsenic Exposure: Methylation Capacity and Beyond

**DOI:** 10.1289/ehp.11276

**Published:** 2008-03

**Authors:** Michael P. Waalkes, Jie Liu

**Affiliations:** Laboratory of Comparative Carcinogenesis, National Cancer Institute at the National Institute of Environmental Health Sciences, National Institutes of Health, Department of Health and Human Services Research Triangle Park, North Carolina, E-mail: waalkes@niehs.nih.gov

Inorganic arsenic (iAs), a toxic metalloid, affects millions of people worldwide, mainly from drinking contaminated water. Arsenic is a human carcinogen that targets skin, lung, bladder, and possibly other sites. iAs and its methylated metabolites readily cross the placenta and reach the fetus [National Research Council (NRC) 1999], producing effects ranging from developmental toxicity to cancer (NRC 1999; Waalkes et al. 2007). Thus, early-life As exposures are drawing escalating health concerns.

In this issue of *Environmental Health Perspectives*, Li et al. (2008) report on iAs metabolism in pregnant Bangladeshi women exposed to iAs through contaminated water. iAs is metabolized to monomethylarsonic acid (MMA) and dimethylarsinic acid (DMA) for urinary excretion; urinary As speciation is widely used to assess As methylation capacity. Arsenic methylation capacity can be influenced by dietary intake of cysteine, methionine, folic acid, niacin, vitamin B12, and choline (Steinmaus et al. 2005), and dietary folic acid supplementation to malnourished arsenicosis patients can decrease As burden by decreasing blood MMA and increasing urinary DMA (Gamble et al. 2007). However, despite poor micronutrient status and high As exposure, the pregnant Bangladeshi women showed remarkably efficient As methylation. The median percentage of urinary DMA (74%) is in the upper range, and MMA (11%) in the lower range of what is commonly seen in urine of individuals from developed counties with much better nutrition (Vahter 2007). Women during childbearing years are more efficient at As methylation than men (Lindberg et al. 2007), particularly during pregnancy. This is likely due to the *de novo* synthesis of choline by the phosphatidylethanolamine methyltransferase (PEMT) pathway (Vahter 2007), which can be up-regulated by estrogen. Thus, the PEMT pathway may function in malnourished pregnant women to increase choline production needed for fetal development, and, perhaps fortuitously for As methylation (Vahter 2007).

The remarkable efficiency of As methylation in malnourished pregnant women could also be an adaptive response to As exposure. Such adaptation might increase As methylation, and thereby excretion, during pregnancy, but perhaps at the expense of later toxicity. Methyl groups from *S*-adenosylmethionine are essential to both As and DNA methylation. DNA methylation status is a well-recognized controlling factor in gene expression. Furthermore, alterations in DNA methylation status are a recognized epigenetic mechanism in As carcinogenesis and are linked with As exposure in various systems (Pilsner et al. 2007; Waalkes et al. 2004). Gestation is a critical period of cell differentiation and genetic programming during development, and a highly sensitive time for initiation of chemical carcinogenesis (Waalkes et al. 2007). Thus, adaptation to As methylation in malnourished pregnant women could impact fetal development via altered gene expression. Activation of the PEMT pathway through estrogen signaling is clearly beneficial for As methylation (Vahter 2007). In contrast, aberrant estrogen signaling is linked with transplacental As carcinogenesis in mice (Waalkes et al. 2004, 2007). Arsenic also has potential synergistic effects with estrogenic carcinogens (Waalkes et al. 2007). Indeed, when *in utero* As exposure is followed by diethylstilbestrol treatment in newborn mice, tumors at multiple sites show synergistic increases in adulthood, including various estrogen sensitive targets (Waalkes et al. 2007).

Recent data show that early-life As exposure has significant impact on human health (Fry et al. 2007; Smith et al. 2006). In newborns from mothers exposed to iAs through contaminated water in Thailand, altered transcript profiles in cord blood include stress-related genes and breast cancer/estrogen-signature genes (Fry et al. 2007), suggesting an adaptive response to As and pointing toward future health issues. Smith et al. (2006) observed a remarkable increase in lung cancer incidence in young adults in Chile who had been exposed to iAs *in utero*; this suggests a human transplacental carcinogenic potential for As. Thus, regardless of the immediate temporal tolerance to As during pregnancy, preventing early-life As exposure of the fetus and improving nutritional status of pregnant women may be critical for the health of future generations.

## Figures and Tables

**Figure:**
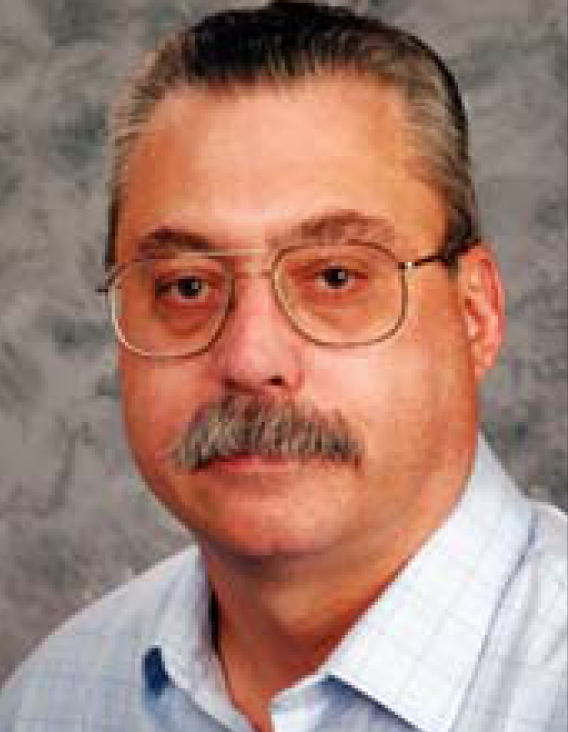
Michael P. Waalkes

**Figure:**
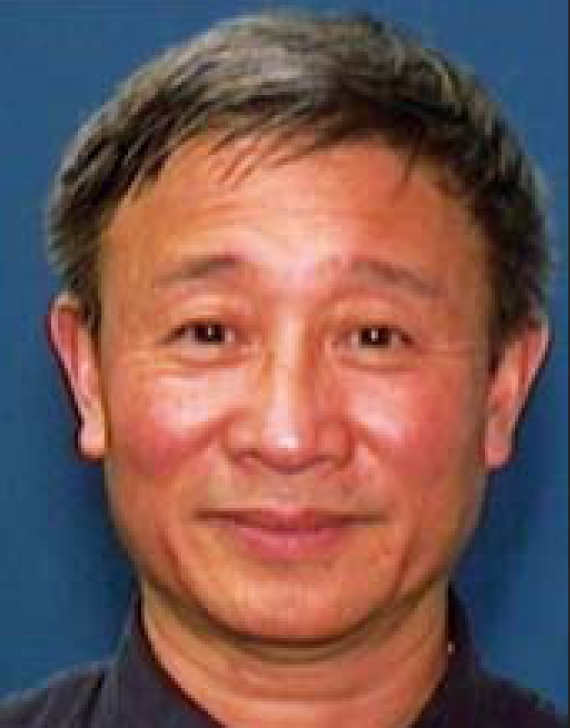
Jie Liu
